# Mind the Gap: Partisan Bias in Justifying Political Violence in the United States

**DOI:** 10.1093/poq/nfag010

**Published:** 2026-03-21

**Authors:** Lars Erik Berntzen, Cornelius Cappelen, Lilliana Mason, Tor Midtbø

**Affiliations:** Associate Professor, Department of Government, University of Bergen, Bergen, Norway; Professor, Department of Comparative Politics, University of Bergen, Bergen, Norway; Professor, Department of Political Science, Johns Hopkins University, Baltimore, MD, US; Professor, Department of Comparative Politics, University of Bergen, Bergen, Norway

## Abstract

Political polarization in America has intensified beyond mere disagreement to what scholars characterize as sectarianism—a condition where partisan identity fundamentally shapes moral judgments. A key marker of sectarianism is asymmetric moral standards for violence, where aggression against political opponents is considered more justified than identical violence targeting one’s own group. Using a survey experiment featuring a realistic political rally scenario, we find compelling evidence in support of such sectarianism: partisan bias in the US extends to evaluations of political violence. By manipulating the partisan affiliations of perpetrators and targets, as well as provocation severity, we find that both Democrats and Republicans exhibit substantial and symmetrical partisan bias. This double standard is particularly pronounced among strong partisans, who are nearly three times more likely to justify violence against the opposition than violence targeting their own party. These results extend sectarianism theory beyond policy preferences to physical violence, suggesting that partisan identity now functions as a powerful perceptual filter that can legitimize political aggression when directed at opponents.

## Introduction

The increasing polarization of American politics has sparked debates about whether partisan identities now resemble the sectarian divisions seen in conflict-ridden societies (Finkel et al. 2020), creating an “us vs. them” mentality where opposing groups are seen as existential threats. While extensive research documents partisan bias in evaluations of policies, candidates, and factual information (Ditto et al. 2019; Banda, Carsey, and Severenchuk 2020), a critical, yet less explored, question is whether this bias extends to justifications of political violence. We argue that sectarian partisanship creates asymmetric moral standards where group loyalty supersedes impartial assessment of political violence.

If partisan sectarianism has taken root, we would therefore expect even acts of physical retaliation violating democratic norms to appear more acceptable when framed as defending one’s group against threats from political opponents. Such asymmetry could pose a direct threat to the democratic principle of resolving conflict through nonviolent competition and heighten the risk of conflict escalation. Yet, despite the importance of this question and growing concerns about political violence, surprisingly little empirical research has directly tested whether partisan bias extends to evaluations of political violence. In fact, to the best of our knowledge, only two studies have explicitly studied this: Kalmoe and Mason (2022) documented partisan bias using a noise blast experiment where Americans were more likely to inflict pain on political opponents than on members of their own party. However, Westwood et al. (2022) find no partisan bias in evaluations of political violence, contradicting what theories of partisan-motivated reasoning would predict. This contradiction may stem from their focus on extreme violence (homicide), resulting in very low overall acceptance rates across respondents, potentially masking partisan differences. The surprising scarcity of empirical research and inconsistent findings raise a critical question: Do Americans truly evaluate political violence impartially, or do scenarios with less extreme violence and varying provocation levels that generate higher baseline acceptance reveal partisan double standards that previous research has missed?

This question is particularly important given the inconsistent findings in the broader literature on attitudes toward political violence. While some surveys estimate that 18–20 percent of Americans condone violence for political goals (Kalmoe and Mason 2022; Prri 2023; Berntzen 2025), others find single-digit support when specifying extreme acts like homicide (Westwood et al. 2022; Holliday et al. 2024).^1^ Following Westwood et al. (2022) and Clifford, Lopez, and Lothamer (2025), these discrepancies reflect two measurement issues: (1) vagueness in questions about “violence”; and (2) the strength of the violent act under consideration. Abstract questions can elicit heterogeneous interpretations (Clifford, Lopez, and Lothamer 2025), whereas focusing on extreme acts like homicide yields uniformly low acceptance rates that can mask partisan differences. We therefore prioritize precision over breadth: we use a fully specified scenario (who does what to whom, under what provocation) and operationalize violence as a concrete, less extreme act (a beating). We extend theories of partisan-motivated reasoning to political violence by manipulating who commits violence, the provocation, and the victim’s identity, holding the violent act constant.

### Theoretical Framework and Hypotheses

We rely on three theoretical perspectives to explain citizens’ justification of political violence: partisan-motivated reasoning (e.g., Mason 2018; Williams 2023; Littvay, McCoy, and Simonovits 2024), the frustration-aggression hypothesis (e.g., Dollard et al. 1939), and intergroup threat theory (e.g., Stephan, Ybarra, and Rios 2015). Our central argument is that sectarian partisanship leads partisans to apply different moral standards when evaluating political violence by their own party versus the opposing party.^2^

Partisan-motivated reasoning theory holds that citizens apply different standards to identical actions depending on whether these actions benefit or harm their political ingroup (Ditto et al. 2019). This reasoning acts as a perceptual filter through which identical behaviors appear justified or condemnable based solely on which party performs them. Under sectarian-like conditions (Finkel et al. 2020), partisans may reflexively defend aggressive acts performed by their own side while condemning identical acts by the opposition. While political aggression can occur without prior provocation (Kalmoe and Mason 2022; Clifford, Lopez, and Lothamer 2025), it often follows perceived provocations that serve as pretexts for retaliation (Berntzen 2025). Two complementary frameworks help explain why provocation intensity shapes violence justification. The frustration-aggression hypothesis posits that blocked goals ignite anger, creating psychological pressure for retaliation (Dollard et al. 1939; Berkowitz 1989). Intergroup threat theory extends this to group contexts, highlighting how perceived threats to a group’s security, values, or capacity to mobilize intensify hostility toward opponents (Stephan and Stephan 2000; Riek, Mania, and Gaertner 2006).

These theories interact in important ways. Partisan-motivated reasoning amplifies the frustration-aggression response specifically when outgroups block ingroup goals, making identical provocations seem more threatening when perpetrated by political opponents. Similarly, intergroup threat perceptions are filtered through partisan lenses, causing each party’s members to perceive equivalent actions as more threatening when performed by the opposition. A further question is whether extreme provocations can “override” partisan bias, temporarily causing Democrats and Republicans to set aside their partisan lenses, or if partisan-motivated reasoning persists even when provocations clearly cross the line.

Finally, we examine whether partisan bias in justifying violence is symmetrical across parties. Research suggests that conservatives exhibit heightened threat sensitivity (Oxley et al. 2008; Hibbing, Smith, and Alford 2014), which may manifest only beyond certain provocation thresholds. While mild provocations might produce similar responses across parties, severe provocations could activate conservatives’ heightened threat systems, potentially leading Republicans to more readily justify violent retaliation specifically in high-threat scenarios. However, studies in other domains often find symmetrical partisan bias (Ditto et al. 2019), leaving this issue unresolved.

Based on the theoretical framework described above (and as specified in our pre-analysis plan), we propose the following hypotheses:

**H1** Introducing partisan labels (inparty or outparty scenarios) will significantly affect respondents’ moral judgments of political violence compared to a neutral control scenario (no partisan labels).

**H2** Respondents will judge violence as more justified when directed against outparty members compared to when directed against inparty members.

**H3** Respondents will perceive violence as more justified when provocation is severe (physical obstruction) compared to mild (shouting and screaming).

**H4** The partisan gap in violence justification (difference between justifying violence against outparty vs. inparty) will decrease in scenarios with severe provocations compared to mild provocations.

**H5** Republicans will show a greater increase in violence justification in response to severe provocations compared to Democrats.

Hypotheses 1 and 2 explore if partisan identity alone biases moral judgments about violence, H1 establishes *whether* partisan cues matter, while H2 establishes the *direction* and *asymmetry* of that bias, crucial for our sectarianism argument.

Hypothesis 3 investigates the effect of provocation severity. Hypotheses 4 and 5 address whether severe provocations moderate partisan bias, potentially in an asymmetric manner.^3^

## Methods and Design

We conducted a 2 × 3 survey experiment (YouGov, *N* = 2,030) in the United States from January 25 to 31, 2024.^4^ See Appendix A for details. The data underlying this article are available in Harvard Dataverse at https://doi.org/10.7910/DVN/C2I1FK. The experimental vignettes present true-to-life scenarios where a political rally is nonviolently disrupted by members of the opposing party. In response, the disruptors are beaten up by attendees from the organizing party. The design manipulates two key variables. The first measures partisan identity, wherein the partisan affiliation of rally organizers (perpetrators) and disruptors (victims) is assigned as either Republican, Democrat, or unspecified (control condition). The second variable measures provocation severity, with the intensity of the nonviolent disruption either mild (shouting and screaming) or severe (physical obstruction).^5^

Our dependent variable measures the perceived justifiability of violence on a seven-point bipolar scale: (1) Completely unjustified, (2) Unjustified, (3) Somewhat unjustified, (4) Neither unjustified nor justified, (5) Somewhat justified, (6) Justified, (7) Completely justified.^6^

Before exposure to the experimental vignettes, respondents were asked to identify their party affiliation and report the strength of their partisan identification. We classify participants into two main categories: strong partisans who explicitly identify as strong Democrats or Republicans, and weak partisans (including those leaning toward a party). The remaining respondents (390) are excluded from further analysis. Thus, all respondents in our analysis are partisans.^7^ The categorization is fully outlined in the Supplementary Material, which also contains a post hoc power analysis.^8^ We employ unweighted data to estimate sample average treatment effects, as random assignment ensures internal validity (Franco et al. 2017).

Table 1 shows the experimental treatments and dependent variable, focusing on the mild provocation scenario.

**Table 1.** Survey design: treatments and outcome measure.

A. Experimental treatments as seen by Republican identifiers (including leaners)		
**1: Inparty, mild provocation** Picture a public rally legally organized by the Democratic Party. As the rally progresses, a group of Republicans try to disrupt the event by shouting and screaming so that it becomes very hard to hear what the speakers are saying. In response, some of the Democrat attendees give them a beating.	**3: Outparty, mild provocation** Picture a public rally legally organized by the Republican Party. As the rally progresses, a group of Democrats try to disrupt the event by shouting and screaming so that it becomes very hard to hear what the speakers are saying. In response, some of the Republican attendees give them a beating.	**5: No party, mild provocation** Picture a public rally legally organized by a political party. As the rally progresses, a group of people try to disrupt the event by shouting and screaming so that it becomes very hard to hear what the speakers are saying. In response, some of the attendees give them a beating.

B. Experimental treatments as seen by Democrat identifiers (including leaners)		
**1: Inparty, mild provocation** Picture a public rally legally organized by the Republican Party. As the rally progresses, a group of Democrats try to disrupt the event by shouting and screaming so that it becomes very hard to hear what the speakers are saying. In response, some of the Republican attendees give them a beating.	**3: Outparty, mild provocation** Picture a public rally legally organized by the Democratic Party. As the rally progresses, a group of Republicans try to disrupt the event by shouting and screaming so that it becomes very hard to hear what the speakers are saying. In response, some of the Democrat attendees give them a beating.	**5: No party, mild provocation** Picture a public rally legally organized by a political party. As the rally progresses, a group of people try to disrupt the event by shouting and screaming so that it becomes very hard to hear what the speakers are saying. In response, some of the attendees give them a beating.

**Question formulation** To what extent do you find it justifiable that the attendees give the disruptors a beating? **Answer scale** (1) Completely unjustified, (2) Unjustified, (3) Somewhat unjustified, (4) Neither unjustified nor justified, (5) Somewhat justified, (6) Justified, (7) Completely justified **Severity of provocation** In the severe provocation scenario (2,4,6), the provocation reads: “… storm through the crowd and physically block the entrance to shut down the rally.”		

In our design, partisan identity and role (victim vs. perpetrator) are perfectly confounded: the ingroup is always either the victim or the perpetrator, never both. Our design does not include scenarios where victim and perpetrator share party affiliation (e.g., Democrat attacking Democrat) or scenarios where the ingroup is uninvolved (e.g., a third party attacking the respondent’s party). This confounding means we cannot determine whether respondents react to partisan identity or to their group’s role as victim versus perpetrator. Still, this mirrors the typical structure of partisan violence, which typically takes the form of intergroup conflict where one’s party is either the target or the aggressor.

Note that some respondents may interpret the provocations (shouting, physical obstruction) as forms of violence themselves (e.g., Hsiao and Radnitz 2021). This can raise the overall mean justification for the retaliatory beating across all conditions. However, because the provocation’s nature and severity are held constant across the key partisan treatments, this does not confound our design’s ability to identify causal effects of partisan bias.

## Results

Across all treatment groups, approximately 19 percent of respondents justify political violence (scoring 5–7 on the answer scale). This aligns with levels found in several other studies of perceptions of political violence (e.g., Kalmoe and Mason 2022; Prri 2023; Wintemute et al. 2024; Berntzen 2025) but exceeds the low level of support found when examining attitudes toward extreme violence like homicide (Westwood et al. 2022; Holliday et al. 2024).

**Figure 1.**
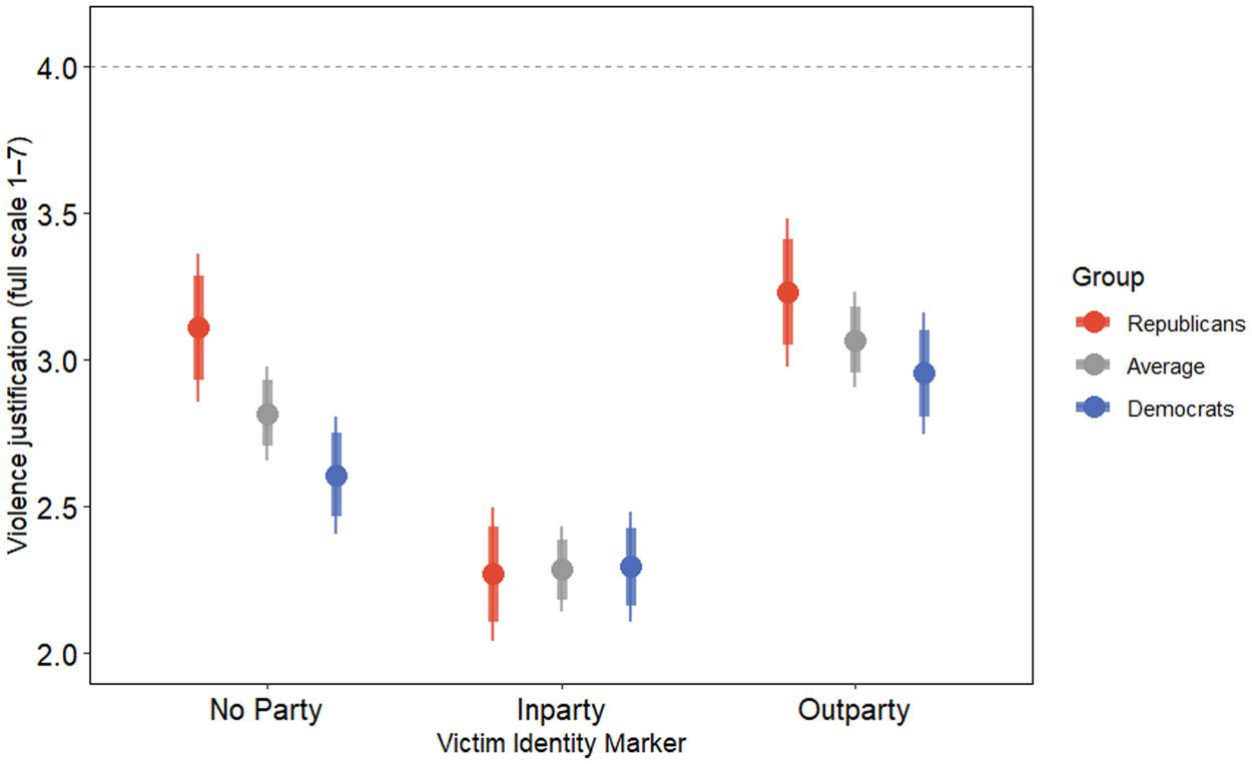
Partisan bias in justifying political violence. Mean violence-justifiability scores with 83.4 percent (thick) and 95 percent (thin) confidence intervals, shown for the control (no party) and the two partisan treatments (inparty, outparty), separately for the combined sample (Democrats and Republicans), Republicans, and Democrats. The dashed horizontal line marks the neutral midpoint (4) on the 1–7 scale to indicate that means remain below the “justified” range.

From figure 1, we observe that the average justification scores between the control group (No party) and the two treatment groups (Inparty/Outparty) differ markedly (and significantly, *p *= 0.001; F-test, Supplementary Material table A5).^9^

When violence targets outgroup members, justification scores increase substantially compared to the control condition, but when it targets ingroup members, justification scores decrease below the control baseline. This suggests that partisan cues do not uniformly increase violence justification, but rather create a split response where violence becomes either more justified (when targeting opponents) or less justified (when targeting one’s own group) than politically neutral violence.

This pattern demonstrates that partisan identity information does not merely shift evaluations in a single direction but fundamentally transforms the moral framework through which identical violent acts are judged, providing strong support for H1. The divergent movement from the neutral baseline when partisan labels are introduced reveals how deeply partisan identity is intertwined with moral reasoning about political violence—creating both permission structures for violence against opponents and heightened protection for ingroup members.

H2 predicts that people will judge violence as more justified when directed against outparty members compared to when directed against inparty members. This hypothesis receives robust support, revealing a stark partisan double standard. Respondents find violence significantly more justified when outparty members are targeted (*mean *= 3.06) than when inparty members are targeted (*mean *= 2.28). This difference of 0.78 points on our seven-point scale (*t *= 7.10) represents a substantial effect (see Supplementary Material table A3; F-test, table A5). Importantly, this gap does not merely reflect subtle attitude differences; it demonstrates fundamentally different moral evaluations of identical violent actions based solely on partisan identity.

**Figure 2.**
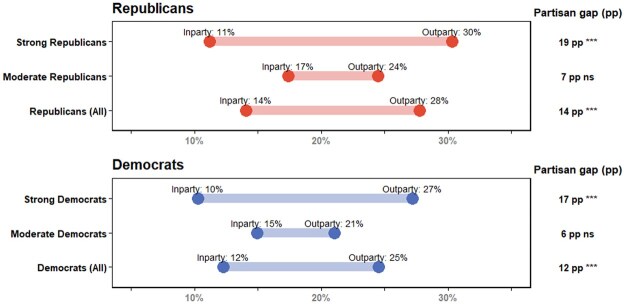
The partisan gap [exploratory]. Percentage of respondents explicitly justifying violence (answered 5–7) by partisan target, with the largest partisan percentage point (pp) gap among strong partisans (Chi-square proportion test). *** *p *< 0.001, ** *p *< 0.01, * *p *< 0.05, ns *p *≥ 0.05.

Exploring this partisan bias further, we focus on the share of the respondents who explicitly condone violence (5–7 on the bipolar answer scale).^10^ This metric identifies individuals who have crossed a meaningful moral threshold: citizens who might potentially support or engage in *actual* political violence. From figure 2 we observe that partisan bias is a phenomenon that appears first and foremost among strong party identifiers: 30 percent of strong Republicans condone violence against Democratic provocateurs, but only 11 percent of them condone violence against Republican provocateurs. This partisan gap is almost identical for strong Democrats: 27 percent justify violence against Republican provocateurs compared to only 10 percent against Democrat provocateurs.

**Figure 3.**
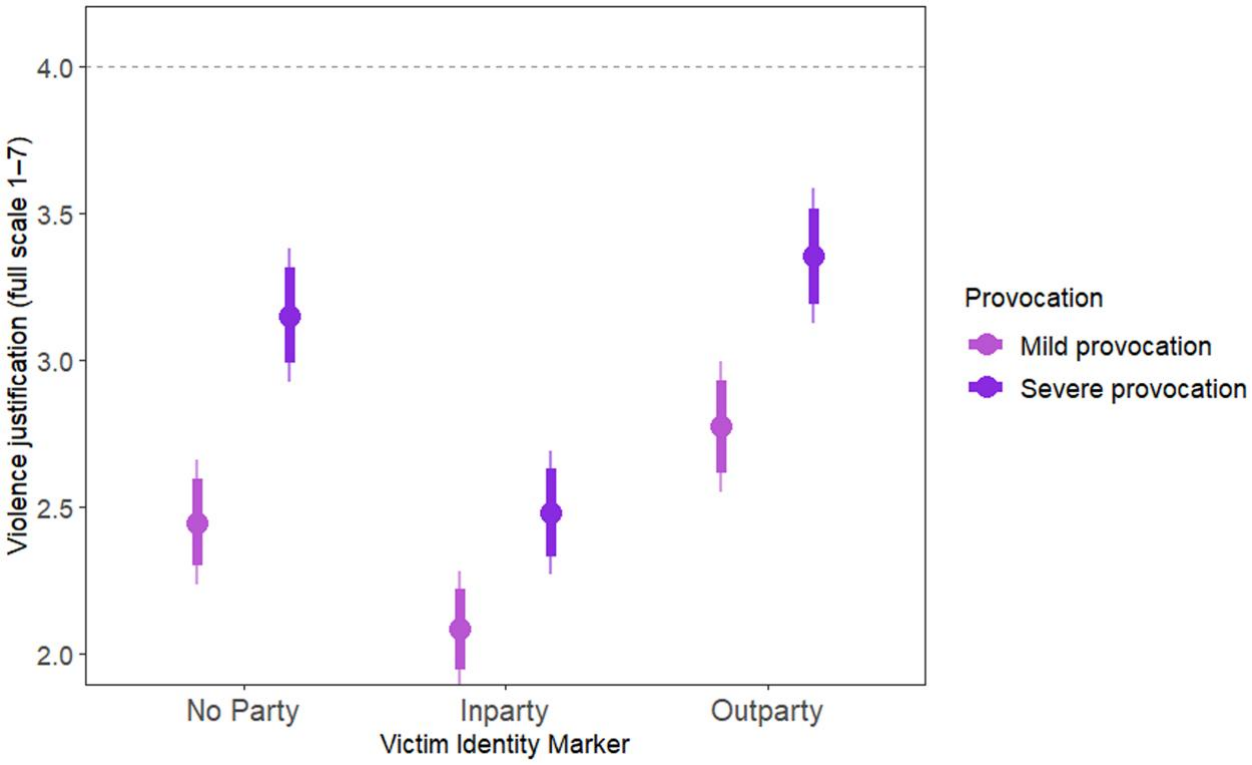
Provocation severity and violence justification. Mean violence justifiability scores with 83.4 percent (thick) and 95 percent (thin) CIs in the six treatment groups for both Republicans and Democrats (average). The dashed horizontal line marks the neutral midpoint (4) on the 1–7 scale to indicate that means remain below the “justified” range.

H3 predicts that the perception of violence is conditioned on the severity of the disruption. This hypothesis is supported. Participants consistently perceive violence as more justified in the more severe disruption scenario (*mean *= 3.00) compared to the milder one (*mean *= 2.44), a substantial difference of 0.56 points on the seven-point scale (*t *= 6.14; Supplementary Material table A4; F-test table A5). This pattern is consistent within and across the partisan identification treatments and “no party” control (see figure 3 below), suggesting that participants were attentive to the scenario details rather than responding based on partisan cues alone.

In the theory section we exploratively hypothesized that the partisan gap in violence justification would be smaller in the severe than in the mild disruption condition (H4), predicting that extreme provocations might “override” partisan filters. Contrary to this prediction, the partisan gap in violence justification remained consistent across both mild (*outparty-inparty difference *= 0.69, *p *= 0.001) and severe provocation scenarios (*outparty-inparty difference *= 0.87, *p *= 0.001). The interaction term in our regression model was nonsignificant (*β* = 0.18, *p *= 0.40; Supplementary Material tables A6–A8). This suggests that partisan bias in violence justification persists with similar strength regardless of provocation severity.

H5 predicts that Republicans will show a greater increase in violence justification in response to severe provocations compared to Democrats. This is not supported by our data. The increase in violence justification from mild to severe provocations was nearly identical for Republicans (*mild-severe difference *= 0.61, *p *= 0.001) and Democrats (*mild-severe difference *= 0.56, *p *= 0.001) (Supplementary Material tables A9–A11).

For full analysis relating to H1–H5, see Supplementary Material section 2. All results are robust to covariate adjustment (Supplementary Material section 5, tables R0–R6).

## Conclusion

Our study provides compelling evidence for the role of partisan bias in shaping perceptions of political violence. Both Democrats and Republicans view identical violent acts as significantly more justified when targeting opposition party members, a finding consistent with theories of partisan-motivated reasoning (Taber and Lodge 2006; Ditto et al. 2019). These partisan biases persist across varying levels of situational provocation. Notably, our mean support levels align with studies using generic violence questions rather than the lower levels found by Westwood et al. (2022), likely because our scenarios include both a provocation and a moderate form of violence (beating) rather than unprovoked or lethal violence. Our findings further demonstrate that partisan identity influences moral judgments in two distinct ways: increasing tolerance for violence against opponents while simultaneously decreasing acceptance of identical violence against one’s own group compared to politically neutral scenarios. The magnitude of this partisan bias is particularly pronounced among strong partisans, suggesting that deeper partisan identification intensifies this moral double standard.

The symmetrical partisan bias we have identified, with both Republicans and Democrats exhibiting similar patterns of moral double standards toward political violence, suggests that moral inconsistency in evaluating violence transcends ideological differences and is rooted in fundamental group identity processes. This partisan double standard in moral evaluation raises serious concerns for democratic stability, as it suggests that partisans may increasingly tolerate or even endorse violence against political opponents while strongly condemning identical actions directed at their own side—a pattern observed in societies that have experienced escalating political conflict.

Our design allows causal inference about attitudes toward violence, but real-world political violence involves additional contextual and social factors that may complicate partisan responses. The findings are specific to violence between ordinary citizens rather than violence targeting political elites, which represents a distinct dimension of political violence that may elicit different responses. Moreover, we cannot generalize these results to all forms of violence, particularly lethal violence, which likely faces higher thresholds for justification than the physical assault described in our scenarios. Furthermore, our experiment does not capture the role of elite rhetoric and cues from political leaders, which can legitimate or condemn violence and significantly influence supporters’ behavior.

Future research should explore whether the symmetrical partisan bias we observed persists across different dimensions by varying the severity of violence, the targets involved, the presence of elite cues, and the wording of the dependent variable (e.g., support, justify, understand, or engage in violence) to assess how partisan identities shape attitudes toward political violence.

## Supplementary Material

nfag010_Supplementary_Data

## Data Availability

Replication data and documentation are available at https://doi.org/10.7910/DVN/C2I1FK.

## Footnotes

1 For related work on support for partisan violence outside the United States, see Berntzen, Kelsall, and Harteveld (2024); Berntzen (2025); and Nai, van Erkel, and Bos (2025). These studies indicate considerable cross-national variation in support for partisan violence in Western democracies.

2 Political violence is typically defined as the deliberate use of power and force to achieve political goals (Krug et al. 2002), including both physical and psychological acts. Following Berntzen (2025), we focus on political violence in its narrow scope: physical violence by adherents of one political faction against another. While psychology literature sometimes distinguishes “aggression” from “violence,” reserving the latter for extreme harm like death (Anderson and Bushman 2002), we follow political science conventions, where the term encompasses a range of physically harmful acts.

3 In addition to these hypotheses, we also preregistered hypotheses concerning exploitation (a “dark personality” trait, measured as willingness to exploit others). Exploitation consistently emerges as the strongest individual-level predictor of violence justification in our regression models (see Supplementary Material section 6 for details).

4 The study was approved by an Ethics Review Board. See the Supplementary Material for this approval and information on sampling procedures. Participants were not provided with formal debriefing, due to the low-risk nature of the study and absence of deception.

5 Interpretations of disruption may vary by event type; US political rallies are generally free public events, not ticketed, which we discuss further in Supplementary Material section 1.

6 Levels of justification may be affected by social desirability bias and expressive responding, as shown by Shair-Rosenfield et al. 2024, who use “understandable” as a follow-up to mitigate such bias. See Supplementary Material section 1 for a discussion.

7 This is standard practice in the literature (e.g., Druckman 2023).

8 See Supplementary Material section 4, tables P1–P2, for the post hoc power and sensitivity analyses, showing strong power for main effects but limited sensitivity for interaction terms.

9 For Republicans, the “No party” control resembles the Outparty treatment, while for Democrats it resembles the Inparty treatment. One possible explanation is that some respondents may have implicitly associated the unlabeled rally with Republicans, though this does not affect the main partisan contrasts we report.

10 See Supplementary Material section 3. We also expand this explorative analysis focusing on those that explicitly justify violence from H2, as discussed here, to H1 and H3.

## Appendix A. AAPOR-Required Disclosure Elements

**First Data Source:** 

Survey data.

**Data Collection Strategy:** 

Survey.

**Research Sponsor and Conductor:** 

YouGov collected the data for the authors.

**Measurement Tools/Instruments:** 

The measurement tool is a web survey. All questions, except the experiment (B), are asked of all the respondents.

*Attention check*

Thank you for participating in our study. It is crucial for the success of our research that you read the questions carefully before answering. We therefore want to check how attentive you are. Please select the number four below. We are very grateful for your participation.

Answer options: 1. _ 2. _ 3._ 4._ 5._

*Partisan identity*

A1. Generally speaking, do you usually think of yourself as a Democrat, a Republican, an Independent, or what?

1. Democrat
2. Republican
3. Independent
4. Some other party
5. Do not want to answer

*PROGRAMING INSTRUCTION: IF ANSWER DEMOCRAT OR REPUBLICAN, ASK, PUTTING IN THE APPROPRIATE PARTY:*

A2. Would you call yourself a strong [Democrat/Republican] or a not very strong [Democrat/Republican]?

1. Strong
2. Not very strong

*PROGRAMING INSTRUCTION: IF ANSWERED INDEPENDENT, SOME OTHER PARTY, OR DO NOT WANT TO ANSWER, ASK:*

A3. If you had to choose, do you think of yourself as closer to the Democratic Party or the Republican Party?

1. Closer to the Democratic Party
2. Closer to the Republican Party
3. Neither

*Experiment—2x3*

*GENERAL INSTRUCTION.*

*Respondents are randomized into one of six groups.*

*PROGRAMING INSTRUCTION FOR GROUPS 3–6 BELOW.*

**IF** a respondent selects **A1** option *1. Democrat* or option *2. Republican* **OR A3** option *1. Closer to the Democratic Party* or option *2. Closer to the Republican Party* **THEN** insert that party in subsequent [inparty-A] brackets.

[outparty-A] is **ALWAYS** the other of the two main parties (Democrat or Republican). For instance, if a respondent picks the Republican Party under A1 or A3 that is [inparty-A], while the Democratic Party is [outparty-A].

**IF** a respondent answers **A3** option *3. Neither* **THEN** they will be randomly assigned to Republican as [inparty-A] and Democrat as [outparty-A] **OR** Democrat as [inparty-A] and Republican as [outparty-A].

Group 1: Control, mild disruption

B1. Picture a public rally legally organized by a political party. As the rally progresses, a group of people try to disrupt the event by shouting and screaming so that it becomes very hard to hear what the speakers are saying. In response, some of the attendees give them a beating.

B1.Q: To what extent do you find it justifiable that the attendees give the disruptors a beating? (1) Completely unjustified, (2) Unjustified, (3) Somewhat unjustified, (4) Neither unjustified nor justified, (5) Somewhat justified, (6) Justified, (7) Completely justified

Group 2: Control, severe disruption

B2. Picture a public rally legally organized by a political party. As the rally attendees are about to enter the venue, a group of people storm through the crowd and physically block the entrance to shut down the rally. In response, some of the attendees give them a beating.

B2.Q: To what extent do you find it justifiable that the attendees give the disruptors a beating? (1) Completely unjustified, (2) Unjustified, (3) Somewhat unjustified, (4) Neither unjustified nor justified, (5) Somewhat justified, (6) Justified, (7) Completely justified

Group 3: Own party violence against opposing party, mild disruption

B3. Picture a public rally legally organized by [inparty-A]. As the rally progresses, a group of [outparty-A] try to disrupt the event by shouting and screaming so that it becomes very hard to hear what the speakers are saying. In response, some of the [inparty-A] attendees give them a beating.

B3.Q: To what extent do you find it justifiable that the [inparty-A] attendees give the [outparty-A] disruptors a beating? (1) Completely unjustified, (2) Unjustified, (3) Somewhat unjustified, (4) Neither unjustified nor justified, (5) Somewhat justified, (6) Justified, (7) Completely justified

Group 4: Own party violence against opposing party, severe disruption

B4. Picture a public rally legally organized by [inparty-A]. As the rally attendees are about to enter the venue, a group of [outparty-A] storm through the crowd and physically block the entrance to shut down the rally. In response, some of the [inparty-A] attendees give them a beating.

B4.Q: To what extent do you find it justifiable that the [inparty-A] attendees give the [outparty-A] disruptors a beating? (1) Completely unjustified, (2) Unjustified, (3) Somewhat unjustified, (4) Neither unjustified nor justified, (5) Somewhat justified, (6) Justified, (7) Completely justified

Group 5: Opposing party violence against own party, mild disruption

B5. Picture a public rally legally organized by [outparty-A]. As the rally progresses, a group of [inparty-A] try to disrupt the event by shouting and screaming so that it becomes very hard to hear what the speakers are saying. In response, some of the [outparty-A] attendees give them a beating.

B5.Q: To what extent do you find it justifiable that the [outparty-A] attendees give the [inparty-A] disruptors a beating? (1) Completely unjustified, (2) Unjustified, (3) Somewhat unjustified, (4) Neither unjustified nor justified, (5) Somewhat justified, (6) Justified, (7) Completely justified

Group 6: Opposing party violence against own party, severe disruption

B6. Picture a public rally legally organized by [outparty-A]. As the rally attendees are about to enter the venue, a group of [inparty-A] storm through the crowd and physically block the entrance to shut down the rally. In response, some of the [outparty-A] attendees give them a beating.

B6.Q: To what extent do you find it justifiable that the [outparty-A] attendees give the [inparty-A] disruptors a beating? (1) Completely unjustified, (2) Unjustified, (3) Somewhat unjustified, (4) Neither unjustified nor justified, (5) Somewhat justified, (6) Justified, (7) Completely justified

*Additional question*

To what extent do you agree with the following statement? I tend to exploit others towards my own end. (1) Strongly disagree, (2) Disagree, (3) Somewhat disagree, (4) Neither agree nor disagree, (5) Somewhat agree, (6) Agree, (7) Strongly agree

**Population Under Study:** 

Survey on the general US adult population. Survey is in English. For details, see Supplementary Material section 2.

**Methods Used to Generate and Recruit the Sample:** 

The company (YouGov) recruits respondents from a large opt-in online panel and uses matched sampling. YouGov draws a random sample from their panel that is larger than the target size and then matches respondents to a sampling frame derived from benchmarks such as the American Community Survey or Current Population Survey. The matching process relies on demographic variables including age, gender, race, education, party identification, and geography. Quotas and poststratification weights are provided. As the YouGov sample is recruited from a nonprobability opt-in panel, conventional margins of sampling error or design effects cannot be computed. YouGov panelists receive incentives through a points-based rewards system, redeemable for gift cards or cash. Note that the use of nonprobability opt-in panels does not guarantee population-level inference. The survey was conducted in the United States.

**Method(s) and Mode(s) of Data Collection:** 

The study was fielded by YouGov using a self-administered Web survey (CAWI) in English. Participants were invited from YouGov’s online panel and completed the survey via browser on desktop or mobile devices. No additional languages or interview modes were used.

**Dates of Data Collection:** 

January 25 to 31, 2024.

**Sample Sizes (By Sampling Frame If More Than One Frame Was Used) and (If Applicable) Discussion of the Precision of the Results:** 

2,030 survey respondents. The survey is a nonprobability online sample (YouGov panel with matched-sample methodology), so a conventional margin of sampling error is not applicable. Because this study uses a nonprobability online sample (YouGov matched panel), we do not report a conventional margin of sampling error. We also do not provide alternative model-based precision measures.

**Whether and How the Data Were Weighted:** 

Analyses were conducted on unweighted data to estimate sample average treatment effects from randomized assignments; no adjustments for design effects due to weighting or clustering were required.

**How the Data Were Processed and Procedures to Ensure Data Quality:** 

The full sample in our survey includes 2,030 respondents. Of these, 119 respondents did not pass the attention check (see questionnaire QA4 at the end of the Supplementary Material), which constituted 5.9 percent of the total sample (D1). Analyses are conducted on the 1,521 attentive partisan respondents retained for the study. Among these, 898 were Democrats (59 percent) and 623 were Republicans (41 percent) (D3). Strong and moderate identifiers were about evenly split, with 55.2 percent strong and 44.8 percent moderate partisans overall (D2).

**IF A PANEL WAS USED: Panel Description:** 

The sample was drawn from YouGov’s recruited online panel. YouGov maintains panel membership through continuous recruitment (web advertising, affiliate networks, and partnerships), double opt-in enrollment, and ongoing profile surveys. Panelists agree to periodic survey invitations and can opt out at any time. YouGov manages participation with quota controls and invitation throttling to limit over-contact, and it monitors data quality via attention checks, response-time flags, and digital fingerprinting; low-quality or inactive members are removed. For this study, YouGov invited eligible US adults to a single, self-administered web survey; there was no longitudinal follow-up, so panel attrition does not apply beyond standard panel maintenance.

**Study Stimuli:** 

Respondents were exposed to text-only vignettes embedded in a 2 × 3 between-subjects survey experiment. Factors were (1) party cue: none, in-party vs. out-party (piped from the respondent’s party ID/lean), and (2) disruption severity: mild (verbal) vs. severe (physical obstruction). Each respondent read exactly one vignette. No images, logos, audio, or video were shown. See elaboration further down, Supplementary Material: The Experiment.

**Dispositions or Response or Participation Rates:** 

The study used YouGov’s nonprobability online access panel. YouGov does not provide study-specific disposition codes (e.g., invitations sent, ineligible, break-offs) required to compute AAPOR participation or cooperation rates.

**Sample Sizes:** 

1,521 respondents included in our analysis; those who failed attention check deleted (119), and furthermore nonpartisan respondents were excluded (390). Full sample was 2,030.

**Measurement and Model Specification:** 

Design and estimands: Randomized 2 × 3 survey experiment estimating sample average treatment effects (SATE). Factors: Party condition (inparty, outparty, no-party control) × Provocation severity (mild, severe).

Main models: OLS on the continuous DV with treatment indicators: Y = β0 + β1(Inparty) + β2(Outparty) + β3(Severe) + β4(Inparty×Severe) + β5(Outparty × Severe) + ε. Party-specific models: estimates reported separately for Democrats and Republicans; combined model includes party subgrouping (stratified presentation). Tests: F-tests for overall treatment effects; planned contrasts for Outparty − Inparty; t-tests for mean differences reported in text and Supplementary Material.

Exploratory models: Logistic (or LPM) for binary “explicitly justify” (5–7) outcome to compute partisan gaps by strength of partisanship. Interaction of provocation severity with party condition to test moderation (H4/H5); interaction terms reported with coefficients and p-values.

Inference: Two-sided tests; 95 percent and 83.4 percent CIs reported (83.4 percent for visual overlap heuristics in figures). Robust (HC) SEs available; results robust to covariate adjustment (Supplementary Material tables R0–R6).

Weighting and design effects: Unweighted analyses (random assignment ensures internal validity for SATE). No clustering or survey weights applied; thus, no design-effect adjustments.

**A General Statement Acknowledging Limitations of the Design and Data Collection:** 

Nonprobability online panel (YouGov matched sample) limits population generalizability; no AAPOR participation/cooperation rates available due to missing disposition counts.

Analyses exclude nonpartisans and inattentive respondents; findings pertain to attentive partisans and may not extend to the broader public.

Vignette design focuses on citizen-on-citizen, nonlethal violence (a beating) following provocation; results may not generalize to lethal violence, elite-targeted violence, or contexts with elite cues.

Partisan identity and actor role (victim vs. perpetrator) are confounded by design, so we cannot isolate whether effects are due to party cues or victim/perpetrator roles.

Web self-administration in English only may introduce coverage and language biases; social desirability and expressive responding may still affect self-reports despite anonymous online mode.
