# Supplementary material for: Mind the Gap: Partisan Bias in Justifying Political Violence in the United States
Source: Public Opin Q. 2026 Mar 21;90(3):758–77. doi: 10.1093/poq/nfag010 (PMC13287516; doi:10.1093/poq/nfag010)
Supplement: nfag010_Supplementary_Data [file nfag010_supplementary_data.pdf]

Supplementary Material.

Mind the gap: Partisan bias in justifying political violence in the United States

Authors:
Lars Erik Berntzen*, Associate Professor, Department of Government, University of Bergen, Bergen, Norway, 0000-0001-5611-3512

Cornelius Cappelen, Professor, Department of Comparative Politics, University of Bergen, Bergen, Norway, 0000-0003-2725-0547

Lilliana Mason, Professor, Department of Political Science, Johns Hopkins University, Baltimore, MD, US, 0000-0002-0305-8246

Tor Midtbø, Professor, Department of Comparative Politics, University of Bergen, Bergen, Norway, 0000-0001-6904-0260

*Corresponding author contact information: Lars Erik Berntzen, Christies gate 17, 5007 Bergen, Norway. Lars.Berntzen@uib.no

Content

1. Descriptives **3**

The Experiment 3

SI Table 1. Pre-registered hypotheses 4

1.1. D1. Sample sizes and retention 5

1.2. D2. Partisanship among retained (Strong/Moderate) 5

1.3. D3. Party family among retained (Democrat/Republican) 5

1.4. D4. Gender distribution among retained 5

1.5. D5A. Age summary among retained 5

1.6. D5B. Age distribution (10-year bins) among retained 5

1.7. D6. Income distribution among retained 6

1.8. D7. Race/ethnicity among retained 6

1.9. D8. Cell counts by party condition 6

1.10. D9. Cell counts by disruption severity 6

1.11. D10. Cell counts by party condition x disruption severity 6

2. Main analyses (A-tables) **7**

2.1. A1. Means and 95% CIs by group (Overall, Republican, Democrat) 7

2.2. A2. H1 Welch: No Party vs Outparty / Inparty (two-sided) 7

2.3. A3. H2 Welch: Outparty > Inparty (one-sided) 7

2.4. A4. H3 Welch: Severe > Mild (overall & within party) 7

2.5. A5. Omnibus ANOVAs and 2-3 factorial (Type III) 8

2.6. A6. H4 OLS coefficients with 95% CIs (Inparty vs Outparty x Severity) 8

2.7 A6b. H4 simple effects (Outparty-Inparty by severity, emmeans) 8

2.8 A7. H4 model fit statistics 8

2.9 A8. H4 Type-III ANOVA 8

2.10 A9. H5 OLS coefficients with 95% CIs (Democrat vs Republican x Severity) 8

2.11 A9b. H5 simple effects (Severe-Mild by party, emmeans) 8

2.12 A10. H5 model fit statistics 9

2.13 A11. H5 Type-III ANOVA 9

3 Exploratory analyses **9**

3.7 E1. H2: One-tailed Chi-square (Outparty - Inparty) 9

3.8 E2. H1: One-tailed Chi-square (No Party - Outparty; No Party - Inparty) 10

3.9 E3. H3: One-tailed Chi-square (Severe - Mild) 10

4 Post hoc power **10**

4.7 P1. Post hoc power and MDES summary (H1-H5) 11

4.8 P2. Post hoc power and MDES by party subgroup (H1-H3) 11

5 Robustness: OLS with covariates (Main Analysis, R-tables) **11**

5.7 R0. H1 baseline OLS (party only; ref = No Party) 12

5.8 R1. H1 OLS + covariates 12

5.9 R2. H2 OLS + covariates (In vs Out) 12

5.10 R3. H3 OLS + covariates (Mild vs Severe) 12

5.11 R4. H4 OLS + covariates (In/Out x Severity) 13

5.12 R5. H5 OLS + covariates (Party family x Severity) 13

5.13 R6. Model fit summary 13

6 Dark personality – hypotheses and analysis **13**

6.7 DARK1. DH1 bivariate OLS (Exploitation → Response) 14

6.8 DARK2. DH1 covariate-adjusted OLS (Exploitation, continuous) 14

6.9 DARK3. DH1 covariate-adjusted OLS (High exploitation 6-7) 15

6.10 DARK4. DH1 standardized OLS (Std. DV & Exploitation) 15

6.11 DARK5. DH2 GAM summary (smooth of exploitation, covariate-adjusted) 15

7 Power of Party Survey **17**

8 IRB approval (screenshot, redacted) **20**

1. Descriptives

We fielded our 2x3 survey experiment in the U.S. from January 25–31, 2024 through YouGov. The company recruits respondents from a large opt-in online panel and uses matched sampling. No human interviewers were used in our survey. YouGov draws a random sample from their panel that is larger than the target size and then match respondents to a sampling frame derived from benchmarks such as the American Community Survey or Current Population Survey. The matching process relies on demographic variables including age, gender, race, education, party identification, and geography. Quotas and post-stratification weights are provided. As the YouGov sample is recruited from a non-probability opt-in panel, conventional margins of sampling error or design effects cannot be computed. YouGov panelists receive incentives through a points-based rewards system, redeemable for gift cards or cash. Note that the use of non-probability opt-in panels does not guarantee population-level inference.

The full sample in our survey includes 2,030 respondents. Of these, 119 respondents did not pass the attention check (see questionnaire QA4 at the end of the Supplementary Material), which constituted 5.9% of the total sample (D1). Analyses are conducted on the 1,521 attentive partisan respondents retained for the study. Among these, 898 were Democrats (59%) and 623 were Republicans (41%) (D3). Strong and moderate identifiers were about evenly split, with 55.2% strong and 44.8% moderate partisans overall (D2).

The demographic portrait of the retained sample is as follows. Age: median = 50 years, with the largest groups in the 45–55 (17.6%) and 65–75 (19.1%) brackets (D5A–B). Gender: Female (51.1%), Male (48.3%), Other (0.6%) (D4). Race/ethnicity: White (70.3%), Black (12.1%), Hispanic (10.7%), Asian (1.7%), Native American (0.9%), two or more races (2.5%), Other (1.2%), and Middle Eastern (0.6%) (D7).

These percentages are mostly in line with 2018 data from the U.S. Census Bureau's American Community Survey (ACS). The ACS reports the following age groups: 18–24 years old (12.08%), 25–34 years old (17.87%), 35–50 years old (24.54%), 51–65 years old (24.88%), and Over 65 (20.65%). Gender data show Male (49.20%) and Female (50.80%), with no option for non-binary or other categories. Racial/ethnic comparisons show White (72.20%), Black (12.70%), Hispanic (18.30%), Asian (5.60%), Native American (<1%), and Other (5.00%).

The Experiment

This experiment uses a 2x3 between-subjects design to explore how partisanship shapes views on political violence. Respondents judge a scenario where a political rally is disrupted, resulting in violence. We vary partisan identity (Republican, Democrat, or unspecified) and disruption severity (mild or severe). In the paper (Table 1), the vignettes are provided as seen by Republican and Democrat respondents.

Our experimental scenarios examining responses to political provocations are grounded in real-world incidents that have occurred across the American political landscape.

The 2016 presidential campaign featured numerous incidents where verbal disruptions at rallies escalated to physical confrontations. To illustrate: At a February 2016 rally in Las Vegas, when a protester verbally disrupted then-candidate Donald Trump, Trump remarked, "I'd like to punch him in the face," adding that in "the old days" protesters would be "carried out on stretchers." At a March 2016 rally in Fayetteville, North Carolina, a Trump supporter physically assaulted a protester who was being escorted out after verbal heckling. The more severe form of provocation in our experiment – physical obstruction – similarly mirrors real-world events. For example, in February 2017, protesters at UC Berkeley physically blocked entrances to a venue where conservative speaker Milo Yiannopoulos was scheduled to appear. When violent confrontations ensued between protesters and would-be attendees, subsequent coverage and public reaction split along partisan lines.

The experiment contrasts mild and severe disruption scenarios to examine how the intensity of provocation affects perceptions of political violence. In the mild disruption treatment, the protesters disrupt the rally by shouting and screaming, making it difficult to hear the speakers. This represents a non-physical, audio-based interference. In contrast, the severe disruption treatment involves protesters physically blocking the entrance to the rally venue, attempting to shut down the event entirely. This scenario represents a more aggressive, physical form of obstruction.

While our vignettes were designed to reflect common features of U.S. political rallies, it is important to note that such rallies are generally free events organized and financed by the campaign. Attendees do not purchase tickets but wait in line, with access limited only by venue

capacity. This makes them more comparable to public gatherings, such as events in a town square, than to private, ticketed occasions like fundraising galas. This contextual detail matters because respondents may interpret the normative boundaries of disruption differently depending on whether the event is seen as a public or private setting. In fact, one might expect that disruptive acts at a private, ticketed event would be judged as more provocative, and thus render violence somewhat more justifiable, than similar disruptions at an open rally. Our choice to use rallies as the experimental setting therefore provides a relatively conservative test: had we framed the scenario in a more "private" context, the observed effects might have been stronger.

For concrete wording of all experimental vignettes, see pre-analysis plan and the survey (titled Power of Party Survey: Deciphering How Partisanship Colors Perceptions of Political Violence), attached at the end of this Supplementary Material.

Our 7-point justifiability scale may be subject to expressive responding and social desirability bias. At a general level, expressive responding can be understood as individuals intentionally providing misinformation as a way of showing support for their political viewpoint (Schaffner and Luks, 2018). In our case, expressive responding could involve insincere exaggeration of violence justification, particularly among strong partisans signaling loyalty. Social desirability bias is the tendency of respondents to underreport socially undesirable traits or behaviors and overreport socially desirable ones to present themselves in a favorable light (Nederhof 1985; Krumpal 2013). In our case, social desirability bias could potentially suppress justification levels across conditions. These two opposing biases may partially offset each other in our data. More importantly, neither bias readily explains our finding that the partisan gap in violence justification varies systematically with provocation levels, suggesting respondents engage substantively with scenario content rather than simply understating socially undesirable responses or exaggerating partisan expression. Note also that online surveys minimize social desirability biases compared to face-to-face and telephone interviewing (e.g., Kreuter et al., 2008; Holbrook and Krosnick, 2010).

SI Table 1. Pre-registered hypotheses[1]

Hypothesis	Description
**H1. Comparative Baseline Hypothesis (Two-Tailed)**	The control conditions and experimental conditions (inparty and outparty aggression scenarios) will significantly differ in how justifiable people find violence.
**H2. Partisan bias hypothesis (One-Tailed)**	Participants will view violence as more justifiable when the outparty commits the provocation (disruption).
**H3. Severity (One-Tailed)**	The use of violence will be perceived as more justifiable in the severe disruption treatment compared to the mild disruption treatment.
**H4. Negation of partisanship**	The relative partisan gap in violence justification is smaller in the severe disruption condition than in the mild disruption condition.
**H5. Differential Response**	Republicans are more likely to find violence justified than Democrats under the severe disruption condition.

[1] Note that we also pre-registered two hypotheses concerning dark personality. We return to this at the end (Supplementary Material: 7. Dark personality), with full analyses and discussion of the findings.

1.1. D1. Sample sizes and retention

Metric	N
Total with non-NA response	2,030
Inattentive (QAttention ≠ 4)	119
Non-partisans (incl. true independents)	423
Retained (attentive partisans)	1,521

1.2. D2. Partisanship among retained (Strong/Moderate)

Category	N	Percent
Strong	839	55.2
Moderate	682	44.8

1.3. D3. Party family among retained (Democrat/Republican)

Category	N	Percent
Democrat	898	59
Republican	623	41

1.4. D4. Gender distribution among retained

Category	N	Percent
Female	777	51.1
Male	735	48.3
Other	9	0.6

1.5. D5A. Age summary among retained

Stat	Value
Min.	18.00
1st Qu.	35.00
Median	50.00
Mean	50.71
3rd Qu.	67.00
Max.	93.00

1.6. D5B. Age distribution (10-year bins) among retained

Category	N	Percent
[65,75)	290	19.1
[45,55)	267	17.6
[25,35)	245	16.1
[35,45)	233	15.3
[55,65)	213	14.0
[75,85)	134	8.8
[18,25)	122	8.0
[85,Inf)	17	1.1

1.7. D6. Income distribution among retained

Category	N	Percent
$80,000 - $99,999	149	9.8
Prefer not to answer	148	9.7
$20,000 - $29,999	133	8.7
$10,000 - $19,999	120	7.9
$30,000 - $39,999	113	7.4
Less than $10,000	111	7.3
$120,000 - $149,999	106	7.0
$40,000 - $49,999	101	6.6
$50,000 - $59,999	96	6.3
$100,000 - $119,999	90	5.9
$70,000 - $79,999	89	5.9
$60,000 - $69,999	80	5.3
$150,000 - $199,999	73	4.8
$200,000 - $249,999	33	2.2
$250,000 - $349,999	33	2.2
Don't know	26	1.7
$350,000 - $499,999	10	0.7
$500,000 or more	10	0.7

1.8. D7. Race/ethnicity among retained

Category	N	Percent
White	1,070	70.3
Black	184	12.1
Hispanic	162	10.7
Two or more races	38	2.5
Asian	26	1.7
Other	18	1.2
Native American	14	0.9
Middle Eastern	9	0.6

1.9. D8. Cell counts by party condition

party_condition	N
No Party	498
Inparty	501
Outparty	522

1.10. D9. Cell counts by disruption severity

disruption_severity	N
Mild	744
Severe	777

1.11. D10. Cell counts by party condition x disruption severity

party_condition	disruption_severity	N
No Party	Mild	238
No Party	Severe	260
Inparty	Mild	248
Inparty	Severe	253

party_condition	disruption_severity	N
Outparty	Mild	258
Outparty	Severe	264

2. Main analyses (A-tables)

Here, we present the analyses corresponding to the hypotheses and results section in the main manuscript. We first report descriptive means and confidence intervals by group (Table A1), followed by Welch tests for H1–H3 (Tables A2–A4) and omnibus F-tests (Table A5). We then turn to factorial ANOVAs and regression models testing interactions for H5–H6 (Tables A6–A11), including model fit and Type-III ANOVA results.

2.1. A1. Means and 95% CIs by group (Overall, Republican, Democrat)

Partisan ID	Provocation	Mean	Lower CI	Upper CI	N	Group
No Party	Mild provocation	2.45	2.23	2.66	238	Overall
No Party	Severe provocation	3.15	2.92	3.38	260	Overall
Outparty	Mild provocation	2.77	2.55	2.99	258	Overall
Outparty	Severe provocation	3.35	3.12	3.58	264	Overall
Inparty	Mild provocation	2.08	1.88	2.28	248	Overall
Inparty	Severe provocation	2.48	2.27	2.69	253	Overall
No Party	Mild provocation	2.78	2.45	3.11	104	Republican
No Party	Severe provocation	3.43	3.05	3.81	104	Republican
Outparty	Mild provocation	2.91	2.57	3.26	115	Republican
Outparty	Severe provocation	3.58	3.21	3.96	101	Republican
Inparty	Mild provocation	2.03	1.73	2.33	103	Republican
Inparty	Severe provocation	2.52	2.17	2.87	96	Republican
No Party	Mild provocation	2.19	1.92	2.46	134	Democrat
No Party	Severe provocation	2.96	2.68	3.25	156	Democrat
Outparty	Mild provocation	2.66	2.36	2.95	143	Democrat
Outparty	Severe provocation	3.21	2.91	3.50	163	Democrat
Inparty	Mild provocation	2.12	1.85	2.38	145	Democrat
Inparty	Severe provocation	2.45	2.18	2.72	157	Democrat

2.2. A2. H1 Welch: No Party vs Outparty / Inparty (two-sided)

Comparison	T-value	df	P-value	CI, lower	CI, upper	Mean (No Party)	Mean (Outparty/Inparty)
No Party vs Outparty	2.181	1,017.930	0.029	0.025	0.479	2.813	3.065
No Party vs Inparty	-4.851	987.894	0.000	-0.747	-0.317	2.813	2.281

2.3. A3. H2 Welch: Outparty > Inparty (one-sided)

Comparison	T-value	df	P-value	CI, lower	CI, upper	Mean (Outparty)	Mean (Inparty)
Outparty vs Inparty	7.089	1,013.23	0.000	0.602	Inf	3.065	2.281

2.4. A4. H3 Welch: Severe > Mild (overall & within party)

Comparison	T-value	df	P-value	CI, lower	CI, upper	Mean (Mild)	Mean (Severe)
Overall: Mild vs Severe	6.140	1,516.10	0.000	0.412	Inf	2.437	3.000
No Party: Mild vs Severe	4.447	495.44	0.000	0.444	Inf	2.445	3.150
Outparty: Mild vs Severe	3.571	519.73	0.000	0.313	Inf	2.771	3.352

Comparison	T-value	df	P-value	CI, lower	CI, upper	Mean (Mild)	Mean (Severe)
Inparty: Mild vs Severe	2.712	497.22	0.003	0.156	Inf	2.081	2.478

2.5. A5. Omnibus ANOVAs and 2-3 factorial (Type III)

Term	df1	df2	F	p_value
party_condition (H1)	2	1,518	25.575	0.000
Inparty vs Outparty (H2)	1	1,021	49.985	0.000
disruption_severity (H3)	1	1,519	37.560	0.000
Type III: (Intercept)	1	1,515	3,598.824	0.000
Type III: party_condition	2	1,515	25.939	0.000
Type III: disruption_severity	1	1,515	38.479	0.000
Type III: party_condition:disruption_severity	2	1,515	0.959	0.384
Type III: Residuals	1,515	1,515		

2.6. A6. H4 OLS coefficients with 95% CIs (Inparty vs Outparty x Severity)

Term	Estimate	Std. Error	t value	p-value	CI lower	CI upper
(Intercept)	2.081	0.112	18.656	0.000	1.862	2.299
party_conditionOutparty	0.691	0.156	4.422	0.000	0.384	0.997
disruption_severitySevere	0.398	0.157	2.534	0.011	0.090	0.706
party_conditionOutparty:disruption_severitySevere	0.183	0.220	0.834	0.404	-0.248	0.614

2.7 A6b. H4 simple effects (Outparty-Inparty by severity, emmeans)

Disruption severity	Contrast	Estimate	SE	df	t value	p-value
Mild	Outparty - Inparty	0.691	0.156	1,019	4.422	0.000
Severe	Outparty - Inparty	0.874	0.155	1,019	5.656	0.000

2.8 A7. H4 model fit statistics

r.squared	adj.r.squared	sigma	statistic	p.value	df	logLik	AIC	BIC	deviance	df.residual	nobs
0.066	0.063	1.756	23.865	0	3	-2,025.744	4,061.488	4,086.141	3,143.264	1,019	1,023

2.9 A8. H4 Type-III ANOVA

Term	Sum Sq	df	F value	p-value
(Intercept)	7,292.357	1	2,364.075	0.000
party_condition	156.450	1	50.719	0.000
disruption_severity	61.194	1	19.838	0.000
party_condition:disruption_severity	2.148	1	0.696	0.404
Residuals	3,143.264	1,019		

2.10 A9. H5 OLS coefficients with 95% CIs (Democrat vs Republican x Severity)

Term	Estimate	Std. Error	t value	p-value	CI lower	CI upper
(Intercept)	2.322	0.087	26.697	0.000	2.152	2.493
party_familyRepublican	0.265	0.132	2.002	0.045	0.005	0.524
disruption_severitySevere	0.556	0.119	4.653	0.000	0.322	0.790
party_familyRepublican:disruption_severitySevere	0.050	0.187	0.267	0.789	-0.316	0.416

2.11 A9b. H5 simple effects (Severe-Mild by party, emmeans)

Party	Contrast	Estimate	SE	df	t value	p-value
Democrat	Severe - Mild	0.556	0.119	1,517	4.653	0.000

Party	Contrast	Estimate	SE	df	t value	p-value
Republican	Severe - Mild	0.606	0.143	1,517	4.228	0.000

2.12 A10. H5 model fit statistics

r.squared	adj.r.squared	sigma	statistic	p.value	df	logLik	AIC	BIC	deviance	df.residual	nobs
0.03	0.028	1.787	15.824	0	3	-3,039.147	6,088.294	6,114.929	4,843.993	1,517	1,521

2.13 A11. H5 Type-III ANOVA

Term	Sum Sq	df	F value	p-value
(Intercept)	11,062.402	1	3,464.428	0.000
party_family	30.784	1	9.641	0.002
disruption_severity	123.811	1	38.774	0.000
party_family:disruption_severity	0.228	1	0.071	0.789
Residuals	4,843.992	1,517		

3 Exploratory analyses

In the main paper we exploratively extend our analysis to those who explicitly justify violence (responses 5–7 on the 7-point scale) for H2. Here, we provide the full Pearson χ^2 one-tailed tests of proportions, not only for H2 but also extended to H1 and H3. Technically, these are a series of repeated 2×2 contingency table analyses where the dependent variable is whether a respondent condones violence (yes/no) and the independent variable is the experimental condition (e.g., inparty vs. outparty, mild vs. severe). Each table yields the observed difference in proportions (our effect estimate in percentage points) and the Pearson χ^2 test statistic. This procedure is the classical large-sample approach to testing independence in categorical data and, in the 2×2 case, is algebraically equivalent to a two-sample z-test for proportions and asymptotically equivalent to the likelihood ratio test from logistic regression. We prefer to report differences in proportions because they are directly interpretable in substantive terms.

For H2, the partisan gap in explicit justification is large and consistent: among attentive partisans overall, the share condoning violence is ~13% higher when the outparty (vs. inparty) is targeted, with the gap ~18% among strong identifiers and smaller but still positive among moderates (see Table E1). Importantly, both Republicans and Democrats exhibit this pattern, especially their strong identifiers; for moderates, intervals sometimes include zero, underscoring weaker partisan sorting at the threshold of explicit violence justification.

For H1, directional contrasts relative to the neutral baseline yield an asymmetric pattern fully consistent with the expectations. Relative to No Party, explicit justification is higher when the outparty is targeted (overall ≈ +4%) and lower when the inparty is targeted (overall ≈ +9% for No Party minus Inparty, i.e., Inparty < No Party). The asymmetry is concentrated among strong identifiers (both contrasts ≈ +9%), while among moderates only the No Party − Inparty difference is robust. By party family, Republicans show a clear No Party − Inparty difference (≈ +13%) and Democrats show both contrasts (≈ +6% each). Full χ^2 tests and bounds are in Table E2.

For H3, severity robustly increases explicit justification. Across all attentive partisans, the Severe − Mild difference is ~11% and remains positive within both strength groups (≈ +8% for strong; ≈ +14% for moderates) and within both party families (Republicans ≈ +11%; Democrats ≈ +11%). These results mirror the mean-difference tests in the main text and indicate that the severity manipulation moves respondents across the morally meaningful threshold of explicit approval (Table E3).

3.7 E1. H2: One-tailed Chi-square (Outparty - Inparty)

Group	Comparison	Estimate	P-value	CI, lower	CI, upper	n1	n2
All Partisans	Outparty - Inparty	0.129	0.000	0.089	1	522	501
Strong Partisans	Outparty - Inparty	0.178	0.000	0.126	1	295	282
Moderate Partisans	Outparty - Inparty	0.064	0.041	0.004	1	227	219
All Republicans	Outparty - Inparty	0.137	0.000	0.073	1	216	199

Group	Comparison	Estimate	P-value	CI, lower	CI, upper	n1	n2
Strong Republicans	Outparty - Inparty	0.191	0.000	0.106	1	122	107
Moderate Republicans	Outparty - Inparty	0.071	0.118	-0.027	1	94	92
All Democrats	Outparty - Inparty	0.123	0.000	0.071	1	306	302
Strong Democrats	Outparty - Inparty	0.168	0.000	0.101	1	173	175
Moderate Democrats	Outparty - Inparty	0.061	0.101	-0.017	1	133	127

3.8 E2. H1: One-tailed Chi-square (No Party - Outparty; No Party - Inparty)

Group	Comparison	Estimate	P-value	CI, lower	CI, upper	n1	n2
All Democrats	No Party - Outparty	-0.062	0.032	-1	-0.007	290	306
All Democrats	No Party - Inparty	0.060	0.021	0.012	1	290	302
All Partisans	No Party - Outparty	-0.040	0.068	-1	0.004	498	522
All Partisans	No Party - Inparty	0.089	0.000	0.050	1	498	501
All Republicans	No Party - Outparty	-0.009	0.422	-1	0.063	208	216
All Republicans	No Party - Inparty	0.129	0.001	0.064	1	208	199
Moderate Democrats	No Party - Outparty	-0.003	0.475	-1	0.079	135	133
Moderate Democrats	No Party - Inparty	0.058	0.111	-0.020	1	135	127
Moderate Partisans	No Party - Outparty	0.021	0.704	-1	0.086	236	227
Moderate Partisans	No Party - Inparty	0.086	0.012	0.024	1	236	219
Moderate Republicans	No Party - Outparty	0.052	0.794	-1	0.157	101	94
Moderate Republicans	No Party - Inparty	0.123	0.022	0.024	1	101	92
Strong Democrats	No Party - Outparty	-0.110	0.008	-1	-0.037	155	173
Strong Democrats	No Party - Inparty	0.058	0.058	-0.003	1	155	175
Strong Partisans	No Party - Outparty	-0.090	0.007	-1	-0.031	262	295
Strong Partisans	No Party - Inparty	0.088	0.002	0.038	1	262	282
Strong Republicans	No Party - Outparty	-0.060	0.154	-1	0.036	107	122
Strong Republicans	No Party - Inparty	0.131	0.006	0.046	1	107	107

3.9 E3. H3: One-tailed Chi-square (Severe - Mild)

Group	Comparison	Estimate	P-value	CI, lower	CI, upper	n1	n2
All Partisans	Severe - Mild	0.106	0.000	0.072	1	777	744
Strong Partisans	Severe - Mild	0.078	0.002	0.034	1	430	409
Moderate Partisans	Severe - Mild	0.139	0.000	0.089	1	347	335
All Republicans	Severe - Mild	0.112	0.000	0.057	1	301	322
Strong Republicans	Severe - Mild	0.103	0.012	0.028	1	163	173
Moderate Republicans	Severe - Mild	0.123	0.007	0.040	1	138	149
All Democrats	Severe - Mild	0.105	0.000	0.064	1	476	422
Strong Democrats	Severe - Mild	0.066	0.028	0.010	1	267	236
Moderate Democrats	Severe - Mild	0.156	0.000	0.093	1	209	186

4 Post hoc power

Below, we show results from the posthoc power analyses (Tables P1 and P2). Effect sizes are reported in raw units on the 1–7 response scale.

As previously reported in this Supplementary Material, the omnibus ANOVAs and the full 2×3 factorial (Table A5) demonstrate highly significant main effects for H1–H3 (all $p < 1e\text{-}09$), and the pairwise contrasts align with these results (Tables A2–A4). Post hoc power

analyses confirm the design has high power (>98%) to detect these main effects (Table P1). For the interaction hypotheses H4 and H5, the observed interaction terms are non-significant in both the coefficient tables and Type-III tests (Tables A6–A11), and power analyses reveal very low power (5.8-13.3%) for the observed small effects, suggesting that any moderation by severity or party family is substantively minor and below the detectable range. Finally, we replicate H1–H3 within Republican and within Democratic subgroups (Table P2), with adequate sensitivity maintained despite smaller cell sizes.

4.7 P1. Post hoc power and MDES summary (H1-H5)

Hypothesis	Model	k (levels)	n_h (harmonic)	df (num)	df (den)	eta2	eta2_partial	f (observed)	Power (%)	f MDES @80%	Approx. MDES (raw 1-7)
H1: party_condition main effect	One-way: party_condition	3	506.78			0.0326		0.184	100.0	0.080	0.159
H2: Outparty vs Inparty	One-way: party_condition	2	511.28			0.0467		0.221	100.0	0.088	0.175
H3: severity main effect	One-way: disruption_severity	2	760.14			0.0241		0.157	100.0	0.072	0.144
H4: (In vs Out) x Severity interaction	LM focal: party_condition:disruption_severity		255.61	1	1,019		0.0007	0.026	13.3	0.088	0.176
H5: (Dem vs Rep) x Severity interaction	LM focal: party_family:disruption_severity		367.03	1	1,517		0.0000	0.007	5.8	0.072	0.144

4.8 P2. Post hoc power and MDES by party subgroup (H1-H3)

Hypothesis	Model	k (levels)	n_h (harmonic)	df (num)	df (den)	eta2	eta2_partial	f (observed)	Power (%)	f MDES @80%	Approx. MDES (raw 1-7)
H1 (Rep): party_condition main effect	One-way: party_condition	3	207.43			0.0525		0.235	100.0	0.125	0.249
H1 (Dem): party_condition main effect	One-way: party_condition	3	299.18			0.0233		0.154	99.0	0.104	0.208
H2 (Rep): Outparty vs Inparty	One-way: party_condition	2	207.15			0.0684		0.271	100.0	0.138	0.276
H2 (Dem): Outparty vs Inparty	One-way: party_condition	2	303.99			0.0337		0.187	99.6	0.114	0.228
H3 (Rep): severity main effect	One-way: disruption_severity	2	311.15			0.0269		0.166	98.5	0.112	0.225
H3 (Dem): severity main effect	One-way: disruption_severity	2	447.38			0.0243		0.158	99.7	0.094	0.188

Comment: Our study was powered to detect moderate main effects across the six primary experimental conditions, each with approximately 300 respondents. This sample size allows us to identify meaningful differences in average responses to the vignettes across conditions. However, the analysis of interaction effects, particularly those involving subgroup comparisons by partisan strength or alignment (e.g., hypotheses H4 and H5, relies on smaller cell sizes (typically 110–160 respondents per subgroup). As a result, our ability to detect small or subtle interaction effects is limited.

5 Robustness: OLS with covariates (Main Analysis, R-tables)

To address potential concerns about omitted variables, we replicated the main analyses with covariate adjustment. Specifically, we included controls for gender, age, race (White vs. Non-White), income group, and partisan strength, in addition to party family and experimental conditions. The results confirm the robustness of our main findings: H1–H3, while the interaction hypotheses (H4, H5) do not reach significance.

5.7 R0. H1 baseline OLS (party only; ref = No Party)

term	estimate	Std. Error	t value	p-value	CI lower	CI upper
(Intercept)	2.788	0.086	32.539	0.000	2.620	2.956
party_condition2	-0.523	0.121	-4.337	0.000	-0.760	-0.286
party_condition3	0.269	0.120	2.246	0.025	0.034	0.504

5.8 R1. H1 OLS + covariates

term	estimate	Std. Error	t value	p-value	CI lower	CI upper
(Intercept)	3.441	0.200	17.222	0.000	3.049	3.833
party_condition2	-0.454	0.115	-3.946	0.000	-0.679	-0.228
party_condition3	0.329	0.114	2.873	0.004	0.104	0.553
partisanship2	-0.028	0.095	-0.291	0.771	-0.214	0.159
disruption_severity2	0.573	0.093	6.141	0.000	0.390	0.757
party_family2	0.356	0.097	3.675	0.000	0.166	0.547
age10	-0.145	0.028	-5.233	0.000	-0.200	-0.091
gender2	-0.680	0.095	-7.161	0.000	-0.866	-0.494
income_group2	-0.117	0.114	-1.024	0.306	-0.341	0.107
income_group3	-0.288	0.130	-2.213	0.027	-0.544	-0.033
race2	0.317	0.107	2.950	0.003	0.106	0.528

5.9 R2. H2 OLS + covariates (In vs Out)

term	estimate	Std. Error	t value	p-value	CI lower	CI upper
(Intercept)	3.109	0.243	12.809	0.000	2.633	3.586
party_condition2	0.776	0.113	6.856	0.000	0.554	0.998
partisanship2	0.063	0.116	0.541	0.589	-0.165	0.290
disruption_severity2	0.507	0.113	4.477	0.000	0.285	0.730
party_family2	0.273	0.118	2.318	0.021	0.042	0.504
age10	-0.165	0.034	-4.887	0.000	-0.231	-0.099
gender2	-0.684	0.115	-5.970	0.000	-0.909	-0.459
income_group2	-0.158	0.138	-1.142	0.254	-0.429	0.113
income_group3	-0.310	0.160	-1.937	0.053	-0.623	0.004
race2	0.393	0.129	3.050	0.002	0.140	0.646

5.10 R3. H3 OLS + covariates (Mild vs Severe)

term	estimate	Std. Error	t value	p-value	CI lower	CI upper
(Intercept)	3.441	0.200	17.222	0.000	3.049	3.833
disruption_severity2	0.573	0.093	6.141	0.000	0.390	0.757
partisanship2	-0.028	0.095	-0.291	0.771	-0.214	0.159
party_condition2	-0.454	0.115	-3.946	0.000	-0.679	-0.228
party_condition3	0.329	0.114	2.873	0.004	0.104	0.553
party_family2	0.356	0.097	3.675	0.000	0.166	0.547
age10	-0.145	0.028	-5.233	0.000	-0.200	-0.091
gender2	-0.680	0.095	-7.161	0.000	-0.866	-0.494
income_group2	-0.117	0.114	-1.024	0.306	-0.341	0.107
income_group3	-0.288	0.130	-2.213	0.027	-0.544	-0.033
race2	0.317	0.107	2.950	0.003	0.106	0.528

5.11 R4. H4 OLS + covariates (In/Out x Severity)

term	estimate	Std. Error	t value	p-value	CI lower	CI upper
(Intercept)	3.167	0.249	12.726	0.000	2.679	3.656
party_condition2	0.656	0.160	4.094	0.000	0.342	0.970
disruption_severity2	0.387	0.161	2.408	0.016	0.072	0.703
partisanship2	0.060	0.116	0.515	0.606	-0.168	0.287
party_family2	0.275	0.118	2.339	0.020	0.044	0.507
age10	-0.165	0.034	-4.874	0.000	-0.231	-0.098
gender2	-0.684	0.115	-5.972	0.000	-0.909	-0.459
income_group2	-0.155	0.138	-1.123	0.262	-0.426	0.116
income_group3	-0.308	0.160	-1.925	0.055	-0.621	0.006
race2	0.393	0.129	3.045	0.002	0.140	0.646
party_condition2:disruption_severity2	0.238	0.226	1.054	0.292	-0.205	0.681

5.12 R5. H5 OLS + covariates (Party family x Severity)

term	estimate	Std. Error	t value	p-value	CI lower	CI upper
(Intercept)	3.501	0.206	17.017	0.000	3.098	3.905
party_family2	0.240	0.136	1.761	0.078	-0.027	0.506
disruption_severity2	0.480	0.121	3.972	0.000	0.243	0.716
partisanship2	-0.029	0.095	-0.301	0.764	-0.215	0.158
party_condition2	-0.453	0.115	-3.944	0.000	-0.679	-0.228
party_condition3	0.331	0.114	2.897	0.004	0.107	0.556
age10	-0.147	0.028	-5.278	0.000	-0.201	-0.092
gender2	-0.682	0.095	-7.189	0.000	-0.869	-0.496
income_group2	-0.120	0.114	-1.049	0.295	-0.344	0.104
income_group3	-0.296	0.130	-2.268	0.023	-0.551	-0.040
race2	0.318	0.107	2.955	0.003	0.107	0.528
party_family2:disruption_severity2	0.233	0.190	1.226	0.221	-0.140	0.607

5.13 R6. Model fit summary

Model	N	R^2	Adj. R^2	Residual SE	Model F	Model df1	Model df2	Model p
R0: party only (No / In / Out)	1,342	0.033	0.031	1.793	22.736	2	1,339	0
H1: party_condition + covariates	1,342	0.132	0.126	1.703	20.277	10	1,331	0
H2: Outparty vs Inparty + covariates	904	0.150	0.141	1.695	17.484	9	894	0
H3: severity + covariates	1,342	0.132	0.126	1.703	20.277	10	1,331	0
H4: (In/Out) x severity + covariates	904	0.151	0.141	1.694	15.849	10	893	0
H5: party_family x severity + covariates	1,342	0.133	0.126	1.703	18.578	11	1,330	0

6 Dark personality – hypotheses and analysis

Recent research highlights the importance of "pre-political" dispositions in shaping attitudes toward political violence. Traits such as relative deprivation, authoritarianism, need for chaos, Manicheanism, trait aggression, and dark personality traits have all been shown to predict political violence (Kalmoe, 2014; Kalmoe & Mason, 2019; Gøtzsche-Astrup, 2021; Berntzen et al., 2024; Landry et al., 2024). Among these, dark personality traits stand out because they also predict actual violent behavior in non-political settings (Hemphill et al., 1998; Pailing et al., 2014; Pechorro et al., 2022; Tetreault et al., 2021). This makes them especially suited for examining the individual-level roots of support for political violence.

In our pre-registration, we specified two hypotheses. These were labelled H4 and H7. To avoid any confusion with the ordering of the other hypotheses above, we call these DH1 and DH2 here. DH1 predicted that respondents with higher dark personality scores would be

more likely to find political violence justifiable than respondents with lower scores. DH2 further hypothesized that the effect of partisanship on justification of violence would be conditioned by respondents' dark personality scores, such that partisan differences would be strongest among those higher in dark personality. [1]

We measured dark personality using the single item "I tend to exploit others toward my own end" from the *Dirty Dozen* scale. This approach is supported by Kajonius et al. (2016), who demonstrated that the core of the Dirty Dozen scale, characterized by manipulative and anti-social traits, can be effectively measured using this single exploitation item, which they term the *Single Item Dirty Dark Dyad* (SIDDD). One-item measures are more vulnerable to measurement error and may underestimate true associations (Bakker & Lelkes, 2018; Engelhardt et al., 2023). Longer instruments capture more nuance, and recent work shows that short-form scales often attenuate the strength of relationships between personality and political outcomes (Osborne et al., 2022). Thus, our results should be viewed as conservative estimates.

Despite this limitation, we find strong support for H4. In the bivariate model, exploitation is a powerful predictor of violence justification ($b = 0.42$, $t = 14.66$, $p < .001$; Table DARK1). The relationship remains highly significant with covariates ($b = 0.37$, $t = 12.81$, $p < .001$; Table DARK2). Importantly, this is on the unstandardized 1–7 scale: a one-point increase in dark personality corresponds to roughly a 0.37-point increase in justification of violence. At the high end, those scoring 6–7 on exploitation average 1.64 points higher in justification than others, a striking difference on a 7-point scale (Table DARK3). Standardized results show the effect is large ($\beta = 0.32$; Table DARK4), on par with or larger than experimental manipulations such as provocation severity and party cues.

By contrast, the evidence does not support DH2. The interaction between partisanship strength and dark personality was not significant, indicating that dark personality does not amplify partisan differences in support for violence. Instead, dark personality exerts a generalized effect across partisan subgroups. This suggests two distinct routes to supporting political violence: one driven by partisan identity (inparty vs. outparty distinctions), and another driven by dispositional acceptance of violence regardless of group context, resembling the patterns described for high "Need for Chaos" individuals (see Table DARK5).

Together, these findings point to a broader conclusion. While our experimental manipulations demonstrate how partisan and contextual cues shape violence acceptance, dark personality represents a powerful individual-level predisposition that pushes attitudes upward across the board. Comparing standardized coefficients suggests that dispositional, partisan, and contextual routes to violence are all consequential, but that dark personality is at least as influential as party cues or disruption severity. This reinforces the importance of integrating personality-based predispositions into future research, which may merit a separate paper with richer data.

6.7 DARK1. DH1 bivariate OLS (Exploitation → Response)

term	estimate	Std. Error	t value	p-value	CI lower	CI upper
(Intercept)	1.803	0.077	23.426	0	1.652	1.954
exploitation	0.424	0.029	14.657	0	0.368	0.481

6.8 DARK2. DH1 covariate-adjusted OLS (Exploitation, continuous)

term	estimate	Std. Error	t value	p-value	CI lower	CI upper
(Intercept)	2.198	0.212	10.366	0.000	1.782	2.614
exploitation	0.369	0.029	12.808	0.000	0.312	0.425
party_condition2	-0.440	0.109	-4.051	0.000	-0.652	-0.227
party_condition3	0.327	0.108	3.028	0.003	0.115	0.539
disruption_severity2	0.565	0.088	6.413	0.000	0.392	0.738
partisanship2	-0.049	0.090	-0.551	0.582	-0.225	0.127
party_family2	0.350	0.092	3.818	0.000	0.170	0.529
age10	-0.073	0.027	-2.710	0.007	-0.125	-0.020
gender2	-0.513	0.091	-5.667	0.000	-0.691	-0.335
income_group2	-0.051	0.108	-0.472	0.637	-0.263	0.161
income_group3	-0.221	0.123	-1.796	0.073	-0.462	0.020

term	estimate	Std. Error	t value	p-value	CI lower	CI upper
race2	0.223	0.102	2.190	0.029	0.023	0.422

6.9 DARK3. DH1 covariate-adjusted OLS (High exploitation 6-7)

term	estimate	Std. Error	t value	p-value	CI lower	CI upper
(Intercept)	3.169	0.198	15.979	0.000	2.780	3.558
exploit_hi_67	1.640	0.207	7.909	0.000	1.233	2.047
party_condition2	-0.453	0.112	-4.031	0.000	-0.674	-0.233
party_condition3	0.325	0.112	2.904	0.004	0.105	0.544
disruption_severity2	0.568	0.091	6.218	0.000	0.389	0.747
partisanship2	-0.075	0.093	-0.803	0.422	-0.257	0.108
party_family2	0.395	0.095	4.159	0.000	0.209	0.581
age10	-0.113	0.027	-4.100	0.000	-0.167	-0.059
gender2	-0.605	0.093	-6.490	0.000	-0.788	-0.422
income_group2	-0.121	0.112	-1.086	0.278	-0.340	0.098
income_group3	-0.295	0.127	-2.321	0.020	-0.545	-0.046
race2	0.319	0.105	3.032	0.002	0.112	0.525

6.10 DARK4. DH1 standardized OLS (Std. DV & Exploitation)

term	estimate	Std. Error	t value	p-value	CI lower	CI upper
(Intercept)	-0.050	0.079	-0.634	0.526	-0.205	0.105
scale(exploitation)	0.323	0.025	12.808	0.000	0.274	0.372
party_condition2	-0.241	0.060	-4.051	0.000	-0.358	-0.124
party_condition3	0.179	0.059	3.028	0.003	0.063	0.296
disruption_severity2	0.310	0.048	6.413	0.000	0.215	0.405
partisanship2	-0.027	0.049	-0.551	0.582	-0.124	0.069
party_family2	0.192	0.050	3.818	0.000	0.093	0.290
scale(age10)	-0.070	0.026	-2.710	0.007	-0.121	-0.019
gender2	-0.282	0.050	-5.667	0.000	-0.379	-0.184
income_group2	-0.028	0.059	-0.472	0.637	-0.144	0.088
income_group3	-0.121	0.068	-1.796	0.073	-0.254	0.011
race2	0.122	0.056	2.190	0.029	0.013	0.232

6.11 DARK5. DH2 GAM summary (smooth of exploitation, covariate-adjusted)

term	edf	ref.df	statistic	p.value
s(exploitation)	1.473	1.780	85.102	0.000
s(age10)	6.558	7.649	2.800	0.006

Supplementary Material references

Bakker, B. N., & Lelkes, Y. (2018). Selling ourselves short? How abbreviated measures of personality change the way we think about personality and politics. *The Journal of Politics, 80*(4), 1311–1325.

Berntzen, L. E., Kelsall, H., & Harteveld, E. (2024). Consequences of affective polarization: Avoidance, intolerance and support for violence in the United Kingdom and Norway. *European Journal of Political Research, 63*(3), 927–949.

Engelhardt, A. M., Feldman, S., & Hetherington, M. J. (2023). Advancing the measurement of authoritarianism. *Political Behavior, 45*(2), 537–560.

Gøtzsche-Astrup, O. (2021). Dark triad, partisanship and violent intentions in the United States. *Personality and Individual Differences, 173,* 110633.

Hemphill, J. F., Hare, R. D., & Wong, S. (1998). Psychopathy and recidivism: A review. *Legal and Criminological Psychology, 3*(1), 139–170.

Holbrook, A. L., & Krosnick, J. A. (2010). Social desirability bias in voter turnout reports: Tests using the item count technique. *Public opinion quarterly*, *74*(1), 37-67.

Kajonius, P. J., Persson, B. N., Rosenberg, P., & Garcia, D. (2016). The (mis)measurement of the Dark Triad Dirty Dozen: Exploitation at the core of the scale. *PeerJ, 4,* e1748.

Kalmoe, N. P. (2014). Fueling the fire: Violent metaphors, trait aggression, and support for political violence. *Political Communication, 31*(4), 545–563.

Schaffner, B. F., & Luks, S. (2018). Misinformation or expressive responding? What an inauguration crowd can tell us about the source of political misinformation in surveys. *Public Opinion Quarterly*, *82*(1), 135-147.

Kalmoe, N. P., & Mason, L. (2019). Lethal mass partisanship: Prevalence, correlates, and electoral contingencies. Paper presented at the *National Capital Area Political Science Association American Politics Meeting*, Washington, DC.

Kalmoe, N. P., & Mason, L. (2022). *Radical American partisanship: Mapping violent hostility, its causes, and the consequences for democracy.* University of Chicago Press.

Kreuter, F., Presser, S., & Tourangeau, R. (2008). Social desirability bias in CATI, IVR, and web surveys: The effects of mode and question sensitivity. *Public Opinion Quarterly, 72*(5), 847–865.

Landry, A. P., Druckman, J. N., & Willer, R. (2024). Need for chaos and dehumanization are robustly associated with support for partisan violence, while political measures are not. *Political Behavior, 46*(4), 2631–2655.

Osborne, D., Satherley, N., & Sibley, C. G. (2022). Personality and ideology: A meta-analysis of the reliable, but non-causal, association between openness and conservatism. In A. Mintz & L. G. Terris (Eds.), *The Oxford handbook of behavioral political science* (online ed.). Oxford University Press.

Pailing, A., Boon, J., & Egan, V. (2014). Personality, the Dark Triad and violence. *Personality and Individual Differences, 67,* 81–86.

Pechorro, P., Curtis, S., DeLisi, M., Maroco, J., & Nunes, C. (2022). Dark triad psychopathy outperforms self-control in predicting antisocial outcomes: A structural equation modeling approach. *European Journal of Investigation in Health, Psychology and Education, 12*(6), 549–562.

Tetreault, C., Bates, E. A., & Bolam, L. T. (2021). How dark personalities perpetrate partner and general aggression in Sweden and the United Kingdom. *Journal of Interpersonal Violence, 36*(9–10), NP4743–NP4767.

Krumpal, I. (2013). Determinants of social desirability bias in sensitive surveys: A literature review. *Quality & Quantity, 47*(4), 2025–2047.

Nederhof, A. J. (1985). Methods of coping with social desirability bias: A review. *European Journal of Social Psychology, 15*(3), 263–280.

7 Power of Party Survey

Deciphering How Partisanship Colors Perceptions of Political Violence

(All questions, except the experiment (B), go to all the respondents).

Attention check.

Thank you for participating in our study. It is crucial for the success of our research that you read the questions carefully before answering. We therefore want to check how attentive you are. Please select the number four below. We are very grateful for your participation.

Answer options: 1. _ 2. _ 3._ 4._ 5._

A Partisan identity.

A1. Generally speaking, do you usually think of yourself as a Democrat, a Republican, an Independent, or what?

1. Democrat
2. Republican
3. Independent
4. Some other party
5. Do not want to answer

PROGRAMING INSTRUCTION: IF ANSWER DEMOCRAT OR REPUBLICAN, ASK, PUTTING IN THE APPROPRIATE PARTY:

A2. Would you call yourself a strong [Democrat/Republican] or a not very strong [Democrat / Republican]?

1. Strong
2. Not very strong

PROGRAMING INSTRUCTION: IF ANSWERED INDEPENDENT, SOME OTHER PARTY, OR DO NOT WANT TO ANSWER, ASK:

A3. If you had to choose, do you think of yourself as closer to the Democratic Party or the Republican Party?

1. Closer to the Democratic Party
2. Closer to the Republican Party
3. Neither

B Experiment – 2x3

GENERAL INSTRUCTION.

Respondents are randomized into one of six groups.

PROGRAMING INSTRUCTION FOR GROUPS 3-6 BELOW.

IF a respondent selects **A1** option *1. Democrat* or option *2. Republican* **OR A3** option *1. Closer to the Democratic Party* or option *2. Closer to the Republican Party* **THEN** insert that party in subsequent [inparty-A] brackets.

[outparty-A] is **ALWAYS** the other of the two main parties (Democrat or Republican). For instance, if a respondent picks The Republican Party under A1 or A3 that is [inparty-A], whilst The Democratic Party is [outparty-A].

IF a respondent answers **A3** option *3. Neither* **THEN** they will be randomly assigned to Republican as [inparty-A] and Democrat as [outparty-A] **OR** Democrat as [inparty-A] and Republican as [outparty-A].

Group 1: *Control, mild disruption*

B1. Picture a public rally legally organized by a political party. As the rally progresses, a group of people try to disrupt the event by shouting and screaming so that it becomes very hard to hear what the speakers are saying. In response, some of the attendees give them a beating.

B1.Q: To what extent do you find it justifiable that the attendees give the disruptors a beating?

(1) Completely unjustified, (2) Unjustified, (3) Somewhat unjustified, (4) Neither unjustified nor justified, (5) Somewhat justified, (6) Justified, (7) Completely justified

Group 2: *Control, severe disruption*

B2. Picture a public rally legally organized by a political party. As the rally attendees are about to enter the venue, a group of people storm through the crowd and physically block the entrance to shut down the rally. In response, some of the attendees give them a beating.

B2.Q: To what extent do you find it justifiable that the attendees give the disruptors a beating?

(1) Completely unjustified, (2) Unjustified, (3) Somewhat unjustified, (4) Neither unjustified nor justified, (5) Somewhat justified, (6) Justified, (7) Completely justified

Group 3: *Own party violence against opposing party, mild disruption*

B3. Picture a public rally legally organized by [inparty-A]. As the rally progresses, a group of [outparty-A] try to disrupt the event by shouting and screaming so that it becomes very hard to hear what the speakers are saying. In response, some of the [inparty-A] attendees give them a beating.

B3.Q: To what extent do you find it justifiable that the [inparty-A] attendees give the [outparty-A] disruptors a beating?

(1) Completely unjustified, (2) Unjustified, (3) Somewhat unjustified, (4) Neither unjustified nor justified, (5) Somewhat justified, (6) Justified, (7) Completely justified

Group 4: *Own party violence against opposing party, severe disruption*

B4. Picture a public rally legally organized by [inparty-A]. As the rally attendees are about to enter the venue, a group of [outparty-A] storm through the crowd and physically block the entrance to shut down the rally. In response, some of the [inparty-A] attendees give them a beating.

B4.Q: To what extent do you find it justifiable that the [inparty-A] attendees give the [outparty-A] disruptors a beating?

(1) Completely unjustified, (2) Unjustified, (3) Somewhat unjustified, (4) Neither unjustified nor justified, (5) Somewhat justified, (6) Justified, (7) Completely justified

Group 5: *Opposing party violence against own party, mild disruption*

B5. Picture a public rally legally organized by [outparty-A]. As the rally progresses, a group of [inparty-A] try to disrupt the event by shouting and screaming so that it becomes very hard to hear what the speakers are saying. In response, some of the [outparty-A] attendees give them a beating.

B5.Q: To what extent do you find it justifiable that the [outparty -A] attendees give the [inparty -A] disruptors a beating?

(1) Completely unjustified, (2) Unjustified, (3) Somewhat unjustified, (4) Neither unjustified nor justified, (5) Somewhat justified, (6) Justified, (7) Completely justified

Group 6: *Opposing party violence against own party, severe disruption*

B6. Picture a public rally legally organized by [outparty-A]. As the rally attendees are about to enter the venue, a group of [inparty-A] storm through the crowd and physically block the entrance to shut down the rally. In response, some of the [outparty-A] attendees give them a beating.

B6.Q: To what extent do you find it justifiable that the [outparty -A] attendees give the [inparty -A] disruptors a beating?

(1) Completely unjustified, (2) Unjustified, (3) Somewhat unjustified, (4) Neither unjustified nor justified, (5) Somewhat justified, (6) Justified, (7) Completely justified

C. Additional question

To what extent do you agree with the following statement?

I tend to exploit others towards my own end.

(1) Strongly disagree, (2) Disagree, (3) Somewhat disagree, (4) Neither agree nor disagree, (5) Somewhat agree, (6) Agree, (7) Strongly agree

8 IRB approval (screenshot)

UNIVERSITY OF BERGEN
Department of Comparative Politics

Cornelius Cappelen

Your ref	Our ref	Date
	2023/17945-ANNPO	21.12.2023

IRB approval - Political affiliation and political violence

**Principal Investigator**	Cornelius Cappelen, Tor Midtbø, Lars Erik Berntzen, all Department of Comparative Politics, and Lilliana Hall Mason, Hohn Hopkins University
**Department**	Department of Comparative Politics
**Protocol Title**	Political affiliation and political violence
**Protocol Number**	2023/17945
**IRB**	Department of Comparative Politics IRB
**Committee Action**	Approved on 20 December 2023

The Department of Comparative Politics IRB has reviewed your research protocol and deemed that it meets the *Guidelines for Research Ethics in the Social Sciences and the Humanities* given by The National Committee for Research Ethics in the Social Sciences and the Humanities (NESH) in 2021.

Your protocol is now approved. You may begin collecting data at any time.

Any changes to the protocol must be approved by the IRB before they are implemented.

Any new information that would affect potential risks to subjects, any problems or adverse reactions must be reported immediately to the IRB.

Yours sincerely,

Kristin Strømsnes
Deputy Head of Department and Director of Research at the Department of Comparative Politics
Chair, Department of Comparative Politics IRB

This document has been electronically approved and therefore has no handwritten signatures.

Telephone +47 55 58 00 00
post@uib.no
Internet www.uib.no
Org. no. 874 789 542

Department of Comparative Politics
Telephone +47 55582175
post@isp.uib.no

Postal address
PO Box 7802
5020 Bergen

Visiting address
Christiesgate 15
Bergen

Case officer
Anna Kristina Polster
+47 +47 21 20 32

page 1 of 1
